# HAGLR promotes neuron differentiation through the miR-130a-3p-MeCP2 axis

**DOI:** 10.1515/med-2021-0301

**Published:** 2021-08-05

**Authors:** Bo Wei, Gui-rong Xiao, Cheng-long Wu, Yi-qin Xu

**Affiliations:** Department of Neurology, Shaoxing People’s Hospital, Shaoxing, Zhejiang, 312000, China

**Keywords:** PD, neuron differentiation, lncRNA-HAGLR, MeCP2, miR-130a-3p

## Abstract

Parkinson’s disease (PD) is a prevalent neurodegenerative disease. Currently, the molecular mechanisms underlying the progressions of PD are not fully understood. The human neuroblastoma cell line SH-SY5Y has been widely used as an *in vitro* model for PD. This study aims to investigate the molecular mechanisms of the non-coding RNA-mediated SH-SY5Y differentiation induced by retinoic acid (RA). By microArray analysis, lncRNA HAGLR was observed to be significantly upregulated during the RA-induced SH-SY5Y differentiation. Silencing HAGLR blocked the RA-induced SH-SY5Y differentiation. Moreover, bioinformatical analysis illustrated that miR-130a-3p contains binding sites for HAGLR. The RNA-pull down assay and luciferase assay demonstrated that HAGLR functioned as a ceRNA of miR-130a-3p in SH-SY5Y cells. Overexpression of miR-130a-3p effectively inhibited SH-SY5Y differentiation. We identified MeCP2, a vital molecule in neuronal diseases, to be a direct target of miR-130a-3p in SH-SY5Y cells by western blot and luciferase assays. The rescue experiments verified that recovery of miR-130a-3p in HAGLR-overexpressing SH-SY5Y cells could successfully overcome the RA-induced SH-SY5Y differentiation by targeting MeCP2. In summary, this study reveals a potential molecular mechanism for the lncRNA-HAGLR-promoted *in vitro* neuron differentiation by targeting the miR-130a-3p-MeCP2 axis, contributing to the understanding of the pathogenesis and progression of PD.

## 1 Introduction

Parkinson’s disease (PD), a neurodegenerative disorder, is one of the most prevalent neurodegenerative diseases worldwide [1]. Symptoms of PD generally develop over years, resulting in socioeconomic burden [1,2]. The molecular mechanisms underlying PD and the effective disease-modifying treatments are still under clinical investigation [3]. The human neuroblastoma SH-SY5Y cell line is an *in vitro* model for PD research since it synthesizes both dopamine (DA) and noradrenaline (NA) [4]. In addition, retinoic acid (RA) has been used to induce the *in vitro* neuronal phenotypes of SH-SY5Y cells by inducing terminal neural differentiation [5]. However, the precise molecular mechanisms for the RA-induced SH-SY5Y differentiation are not fully understood.

lncRNAs, which are non-coding RNAs with relatively larger sizes (>200 nucleotides), have been extensively studied as important regulators for diverse diseases [6,7]. Accumulating evidence revealed that lncRNAs function by specifically binding to DNA, RNA, or proteins to regulate gene expressions [8]. Moreover, studies uncovered that lncRNAs act as competitive endogenous RNAs (ceRNA) of miRNAs by sponging them to de-repress the expressions of miRNA target genes [9]. lncRNA HAGLR (HOXD antisense growth-associated long non-coding RNA) has been reported to be positively associated with various diseases [10,11,12,13]. Currently, the biological roles of HAGLR in neuronal diseases such as PD have not been elucidated.

Methyl CpG binding protein 2 (MeCP2), which specifically binds to methylated cytosines on DNA, is a member of the methyl-CpG-binding domain (MBD) protein family [14]. Studies reported that MeCP2 was the most abundant molecule in the adult brain and tightly correlated with diverse neuronal processes during neurodevelopment [15]. Moreover, mutations in the MeCP2 gene cause the Rett syndrome (RTT), which is a neurologic condition affecting young girls [16] and lead to the MeCP2 duplication syndrome (MDS) [17]. These studies suggest MeCP2 is an important diagnostic biomarker and therapeutic target for neuronal diseases. However, the precise roles and molecular mechanisms of MeCP2 in PD are still under investigation.

In this study, we investigated the biological functions of HAGLR and MeCP2 during the RA-induced neuron differentiation of the SH-SY5Y neuroblastoma cell line. From lncRNA-microarray analysis, for the first time, we discovered HAGLR was upregulated from the *in vitro* neuronal differentiation model. The lncRNA–miRNA ceRNA network and the targets of miRNA will be identified.

## 2 Materials and methods

### 2.1 Cell culture and treatment

Human SH-SY5Y and SK-N-MC cells were cultured in Dulbecco’s modified Eagle’s medium/ F12 medium (DMEM/F12) (Thermo Fisher Scientific, Shanghai, China) supplemented with 10% fetal bovine serum (FBS) (Thermo Fisher Scientific, Shanghai, China) with 100 units/mL penicillin and 100 μg/mL streptomycin (Thermo Fisher Scientific, Shanghai, China) at 37°C in a humidified atmosphere with 5% CO_2_. RA induction was performed as previously described [18]. Cells were treated with 5 µM RA for 3–7 days. Morphological changes were monitored under a brightfield microscope. RA was purchased from Sigma-Aldrich (Shanghai, China). Mouse anti-MeCP2 (#M6818) and β-actin antibodies (#A5441) were purchased from Sigma-Aldrich (Shanghai, China).

### 2.2 Transfections of shRNA, miRNA, and plasmid DNA

Transfections were performed using Lipofectamine 2000 (Invitrogen, Carlsbad, CA, USA) according to the manufacturer’s protocols. SH-SY5Y cells (1 × 10^6^) were plated in 60 mm wells for 24 h. siRNA, miRNAs, shRNA, and their negative controls were synthesized by GenePharma Inc. (Shanghai, China) and transfected at 50 nM for 48 h. Plasmid DNA was transfected at 2 µg/well for 48 h. Cells were then treated for 48 h with RA. Experiments were conducted in triplicate and repeated at least three times.

### 2.3 lncRNA–miRNA and miRNA–mRNA interaction analysis

The HAGLR-miR-130a-3p and miR-130a-3p-MeCP2 3′UTR bindings were predicted from Targetscan.org and starBase of ENCORI http://starbase.sysu.edu.cn/ according to previous reports [19]. The online non-coding RNA service predicts binging of miRNA with lncRNAs or mRNA targets of miRNA by searching for the presence of conserved 8mer, 7mer, and 6mer sites that match the seed region of each miRNA. Prediction of starBase was from 108 CLIP-Seq (PAR-CLIP, HITS-CLIP, iCLIP, and CLASH) data sets generated by 37 independent studies.

### 2.4 Quantitative RT-PCR

Total RNA was extracted from SH-SY5Y cells using the RNeasy Mini Kit (Qiagen, Shanghai, China) according to the manufacturer’s instructions. The quality and concentration of RNA samples were determined using a Nanodrop 2000 spectrophotometer (Thermo Fisher Scientific, Shanghai, China). Total RNA was treated with DNase I, followed by reverse transcription into cDNA using the PrimeScript RT Master Mix (TaKaRa, Dalian, China) according to the manufacturer’s instructions. The qRT-PCRs were performed using the SYBR Green qPCR Master Mix (ThermoFisher Scientific, Waltham, MA, USA) in an ABI PRISM 7500 Real-Time System. A total of 40 amplification cycles were applied with the following settings: denaturation at 95°C for 15 s, annealing at 58°C for 30 s, and extension at 72°C for 42 s. The relative gene expression was determined by the 2^−ΔΔ*C*t^ method. β-Actin was an internal control for lncRNA and MeCP2 mRNA detection, and U6 was an internal control for miRNA detection. Experiments were performed in triplicate and repeated three times.

### 2.5 RNA pull-down assay

The RNA pull-down assay was performed as described previously. Briefly, biotin-labeled scramble, sense, or antisense lncRNA HAGLR DNA oligomers (RiboBio) were incubated with the SH-SY5Y cell lysate followed by incubation for 1 h; then streptavidin-coupled agarose beads (Thermo Fisher Scientific, Shanghai, China) were added to pull down the RNA–RNA complexes. The miR-130a-3p expressions were analyzed from the RNA–RNA complexes by quantitative RT-PCR (qRT-PCR). Experiments were repeated three times.

### 2.6 Luciferase assay

The wild-type (WT) and mutated (Mut) HAGLR or MeCP2 3′UTR were amplified and cloned into the pGL3-control luciferase reporter vector system (Promega, Madison, WI, USA). SH-SY5Y cells were co-transfected with control miRNA or miR-130a-3p with WT or Mut HAGLR or MeCP2 3′UTR using Lipofectamine 2000 (Invitrogen, Carlsbad, CA, USA) for 72 h. Luciferase activity was measured using a Dual-Luciferase reporter assay system (Promega, Madison, WI, USA) according to the protocol from the kit. The experiment was performed in triplicate and repeated three times.

### 2.7 Microarray analysis

Total RNA was extracted from SH-SY5Y cells using the RNeasy Mini Kit (Qiagen, Shanghai, China) according to the manufacturer’s instructions. RNA was quantified using a Nanodrop 2000 spectrophotometer (Thermo Fisher Scientific, Shanghai, China). Integrities of RNA samples were assessed using an Agilent Bioanalyzer 2100 (Agilent Technologies, Santa Clara, CA, USA). RNA was amplified and labeled. The Cy3-labeled complementary RNA was hybridized using the Gene Expression Hybridization Kit (Agilent Technologies, Santa Clara, CA, USA). Slides were scanned using an Agilent Microarray Scanner (Agilent Technologies, Santa Clara, CA, USA). The MicroArray results were analyzed using Agilent Feature Extraction software (Agilent Technologies, Inc.) and GeneSpring GX v11.5.1 software (Agilent Technologies, Inc.).

### 2.8 Western blot

Proteins from SH-SY5Y cells were extracted using RIPA buffer (ThermoFisher Scientific, Inc.) with a 1× protease inhibitor cocktail (Thermo Fisher Scientific Inc.). After 20 min incubation on ice, lysates were centrifuged at 10,000 *g* for 10 min at 4°C. An equal amount of protein from each sample was separated by SDS-PAGE and transferred to polyvinylidene difluoride membranes (PVDFs) (Millipore, Bedford, MA, USA) followed by blockage for 1 h at room temperature with 4% bovine serum albumin (BSA). Membranes were incubated with primary antibodies at 1:1,000 overnight at 4°C. After washing with phosphate buffered saline with tween 20 (PBST), membranes were incubated with secondary antibody (1:3,000) for 1 h at room temperature. Blots were visualized with enhanced chemiluminescence (Millipore, Bedford, MA, USA). β-Actin was used as a loading control. Experiments were repeated three times.

### 2.9 Statistical analysis

Data were analyzed using Prism version 7.0 (GraphPad Software, La Jolla, CA, USA). Data were expressed as mean ± standard deviation (SD). Experiments were repeated three times. Independent unpaired Student’s *t*-test was applied to compare differences between two groups. Values of *p* < 0.05 were considered statistically significant.

## 3 Results

### 3.1 HAGLR is upregulated during the RA-induced neuron differentiation

**Figure 1:**
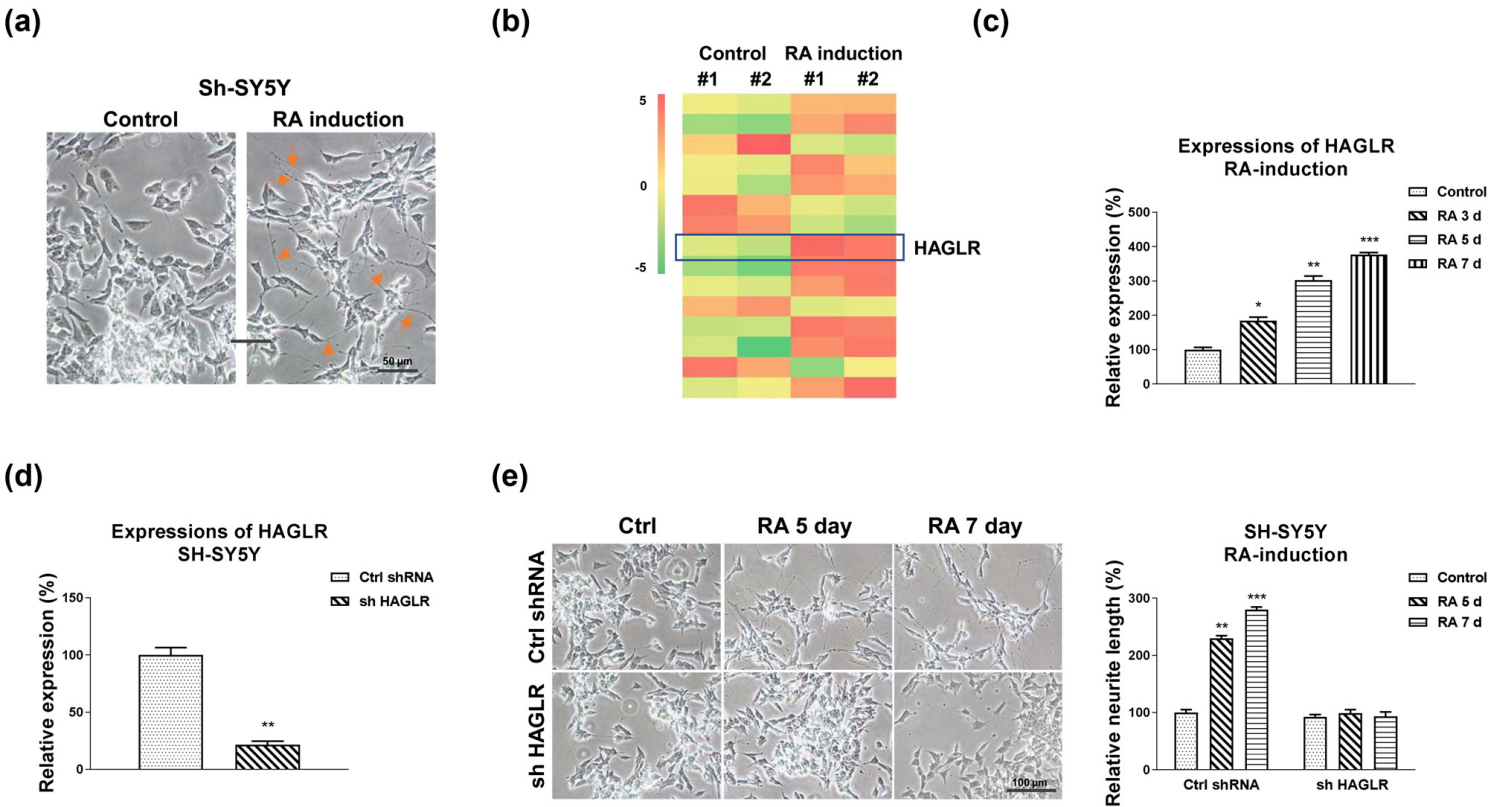
Roles of HAGLR in RA-induced neuron differentiation. (a) Effects of RA treatment on cell morphology. SH-SY5Y cells were treated with 5 µM RA for 5 days. (b) SH-SY5Y cells were treated with RA using the above protocol. RNAs were isolated and lncRNA-microArray analysis was performed in duplicate. (c) SH-SY5Y cells were treated with 5 µM RA for 3, 5, and 7 days. HAGLR expressions were examined by qRT-PCR. (d) SH-SY5Y cells were transfected with control shRNA or HAGLR shRNA for 48 h, and expression of HAGLR was determined by qRT-PCR. (e) SH-SY5Y cells were transfected with control shRNA or HAGLR shRNA for 48 h, followed by treatments with 5 µM RA for 5 and 7 days. Cell morphologies were examined under a microscope, and the neurite length was measured. *p < 0.05; **p < 0.01; and ***p < 0.001.

**Figure S1:**
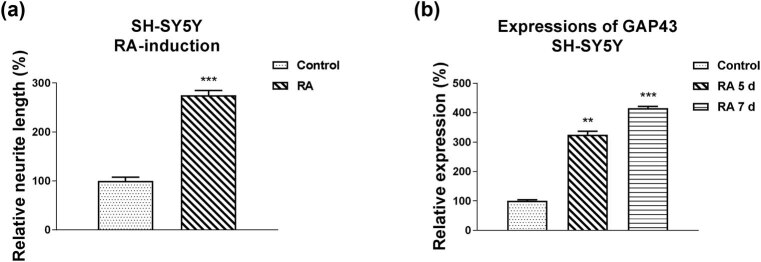
Effects of retinoic acid treatment on neuron differentiation. (a) SH-SY5Y cells were treated with RA at 5 µM for 5 days, the relative neurite length was calculated. (b) Expressions of GAP43 in SH-SY5Y cells under RA treatments at 5 days and 7 days were determined by qRT-PCR. **, *p* < 0.01; ***, *p* < 0.001.

**Figure S2:**
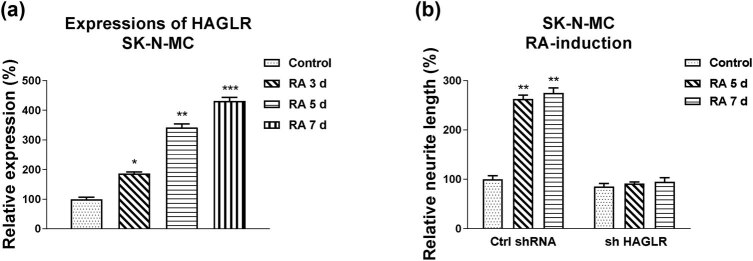
Effects of retinoic acid treatment on SK-N-MC cells. (a) Expressions of HAGLR in SK-N-MC cells under RA treatments for 3, 5 and 7 days were determined by qRT-PCR. (b) The relative neurite length was calculated in SK-N-MC cells without or with HAGLR silencing under RA treatments for 5 and 7 days. *, *p* < 0.05; **, *p* < 0.01; ***, *p* < 0.001.

We evaluated the cellular mechanisms of neuron differentiation using RA-induction of SH-SY5Y cells as an *in vitro* model, which has been widely used in PD research [4]. The results showed that the RA treatment (5 μM) of SH-SY5Y cells for 4 days induced remarkable morphologic differentiation with neurite extension compared with control cells that showed no morphological differentiation (Figure 1a and Figure S1a). Moreover, the neuron differentiation marker, GAP43, was induced under RA treatments (Figure S1b), indicating successful neuronal induction by RA. Accumulating studies revealed that non-coding RNAs play vital roles during neuron differentiation [7,8]. To investigate the molecular mechanisms, we performed a lncRNA microArray analysis to profile lncRNA expressions using SH-SY5Y cells with or without RA induction. Among the differentially expressed lncRNAs in SH-SY5Y cells with RA induction, we observed that lncRNA HAGLR was significantly upregulated by the RA treatment (Figure 1b), suggesting that HALGR is positively associated with neuron differentiation. The results from microArray were further validated by qRT-PCR in SH-SY5Y cells with RA induction at 3, 5, and 7 days. In addition, the RA-induced HAGLR upregulation at 5 and 7 days was observed in another neuroblastoma cell line, SK-N-MC (Figure S2a). To assess the biological functions of HAGLR in neuron differentiation, HAGLR was knocked down in SH-SY5Y cells (Figure 1d). As we expected, SH-SY5Y and SK-N-MC cells with HAGLR silencing displayed little response to RA treatments compared with the control shRNA transfected cells (Figure 1e and Figure S2b). Taken together, these results consistently demonstrated that HAGLR promotes neuron differentiation from an RA-induced *in vitro* SH-SY5Y model.

### 3.2 HAGLR sponges miR-130a-3p as a ceRNA in SH-SY5Y cells

**Figure 2:**
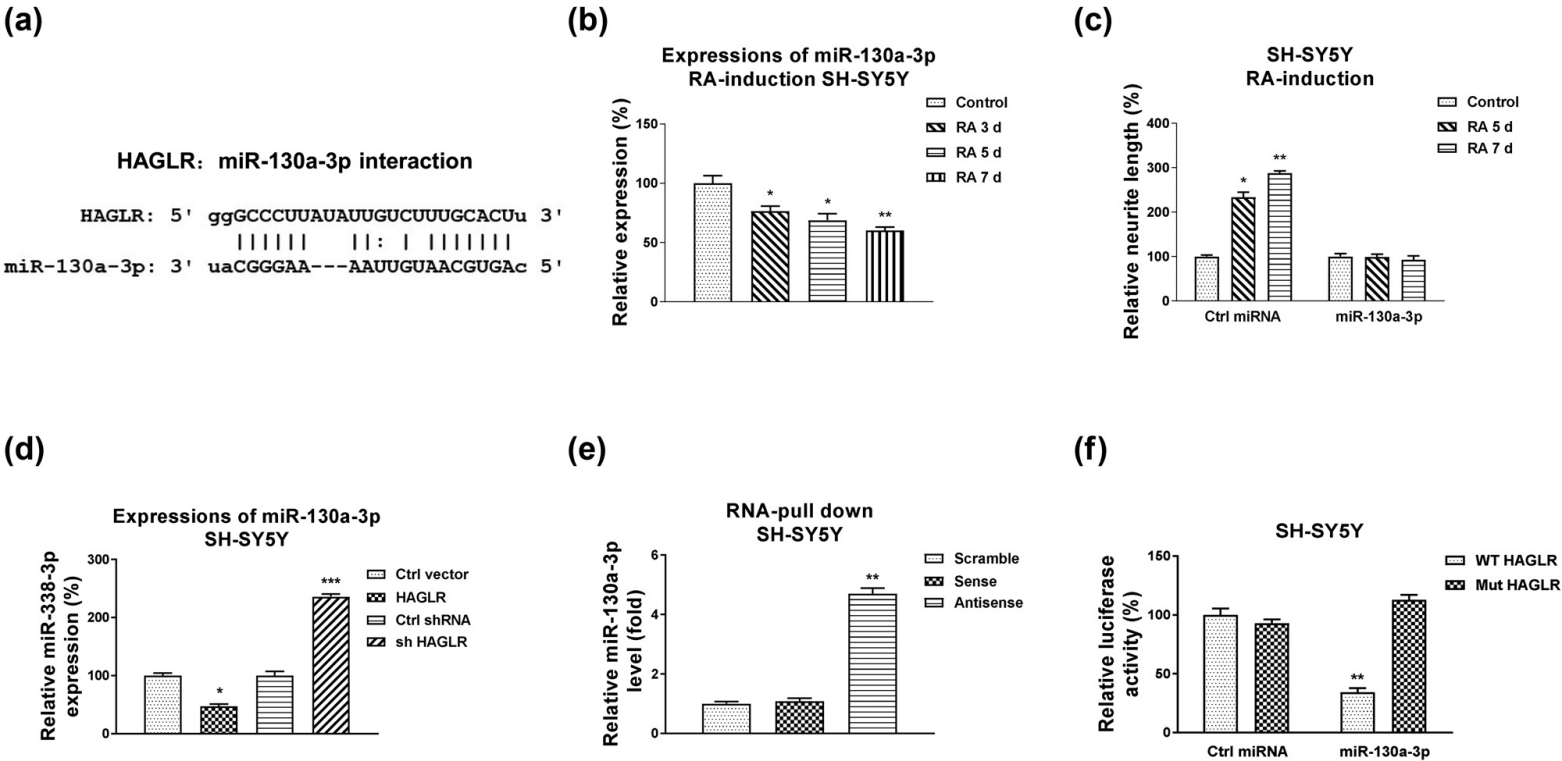
HAGLR sponges miR-130a-3p to negatively regulate its expression. (a) Bioinformatics analysis of potential binding of HAGLR on miR-130a-3p. (b) SH-SY5Y cells were treated with 5 µM RA for 3, 5, and 7 days. miR-130a-3p expressions were examined by qRT-PCR. (c) SH-SY5Y cells were transfected with control miRNA or miR-130a-3p for 48 h, followed by treatments with 5 µM RA for 5 and 7 days. Cell morphologies were examined under a microscope, and the neurite length was measured. (d) SH-SY5Y cells were transfected with control, the HAGLR overexpression plasmid, or HAGLR shRNA for 48 h; miR-130a-3p expressions were examined by qRT-PCR. (e) SH-SY5Y cell lysates were incubated with biotin-labeled scramble, sense, or antisense HAGLR DNA probes. Biotin pull-down assay was performed, and miR-130a-3p expression was assessed by qRT-PCR. (f) Dual-luciferase reporter assay was performed in SH-SY5Y cells with co-transfection of WT-HAGLR or Mut-HAGLR with control miRNA, or miR-130a-3p. *p < 0.05; **p < 0.01; and ***p < 0.001.

**Figure S3:**
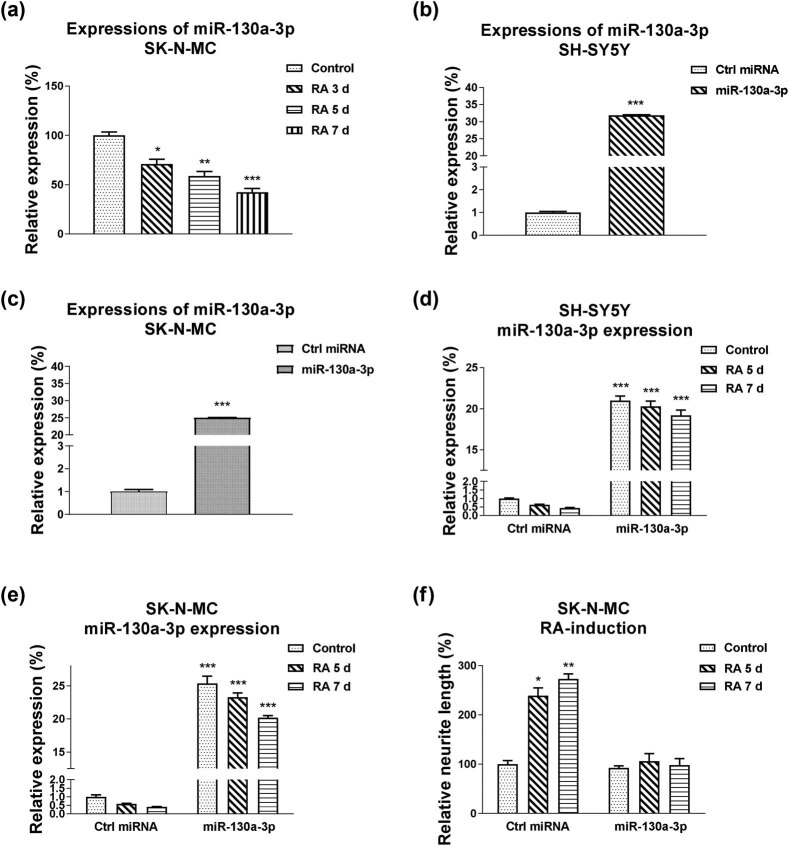
miR-130a-3p suppresses RA-induced neuron differentiation. (a) Expressions of miR-130a-3p in SK-N-MC cells under RA treatments at 3, 5 and 7 days were determined by qRT-PCR. (b) Effects of miR-130a-3p overexpression in SH-SY5Y and (c) SK-N-MC cells. (d) Detection of miR-130a-3p expressions in SH-SY5Y and (e) SK-N-MC cells without or with miR-130a-3p overexpression under RA treatments for 5 and 7 days. (f) The relative neurite length was calculated in SK-N-MC cells without or with miR-130a-3p overexpression under RA treatments for 5 and 7 days. *, *p* < 0.05; **, *p* < 0.01; ***, *p* < 0.001.

We next explored the molecular mechanisms underlying the HAGLR-promoted neuron differentiation. Previous studies uncovered that lncRNAs function by interfering with target miRNAs as molecular sponges [9]. The downregulation of miRNAs by lncRNA leads to de-repression of their target mRNAs [20]. To identify the potential miRNA targets of HAGLR, we analyzed the HAGLR–miRNA interaction through the non-coding RNA database, starBase2.0. We observed miR-130a-3p, which plays an important role during neuron development and neuronal diseases [21], contains HAGLR binding sites (Figure 2a). We then evaluated the functional roles of miR-130a-3p during RA-induced neuron differentiation. qPCR results remarkedly showed a reverse phenotype between HAGLR and miR-130a-3p, which was significantly downregulated in SH-SY5Y and SK-N-MC cells with RA induction at 3, 5, and 7 days (Figure 2b and Figure S3a). Moreover, although RA treatments suppressed miR-130a-3p expressions, exogenous overexpression of miR-130a-3p (Figure S3b and c) rendered cells to maintain relatively high miR-130a-3p expression levels (Figure S3d and e). Expectedly, SH-SY5Y and SK-N-MC cells with exogenous overexpression of miR-130a-3p showed no effective morphological changes in the neurite extension under RA treatments (Figure 2c and Figure S3f). To assess whether HAGLR downregulates miR-130a-3p, HAGLR was overexpressed or silenced in SH-SY5Y cells. Expectedly, overexpression of HAGLR effectively blocked the miR-130a-3p expressions, while knockdown of HAGLR significantly upregulated miR-130a-3p expressions (Figure 2d). To validate the binding of miR-130a-3p on HAGLR, we performed an RNA pull-down assay. The biotin-labeled scramble, sense, or antisense DNA probe of HAGLR was incubated with SH-SY5Y cell lysates. The qRT-PCR results demonstrated that only the antisense HAGLR DNA probe pulled down the enriched miR-130a-3p (Figure 2e). The endogenous miR-130a-3p could not be effectively pulled down by the scramble or sense HAGLR probe (Figure 2e), suggesting that HAGLR specifically interacts with miR-130a-3p in neuroblastoma cells. To validate whether HAGLR directly binds on the seeding region of miR-130a-3p, SH-SY5Y cells were co-transfected with a luciferase vector containing the wild-type HAGLR (WT-HAGLR) or the binding site mutant HAGLR (Mut-HAGLR) and control miRNA or miR-130a-3p. Luciferase activity of cells with WT-HAGLR and miR-130a-3p co-transfection was significantly suppressed compared with that from Mut-HAGLR and miRNA-130a-3p co-transfection (Figure 2f). In summary, these results demonstrated that HAGLR sponges miR-130a-3p by forming a ceRNA network.

### 3.3 miR-130a-3p directly targets MeCP2 and inhibits the RA-induced neuron differentiation

**Figure 3:**
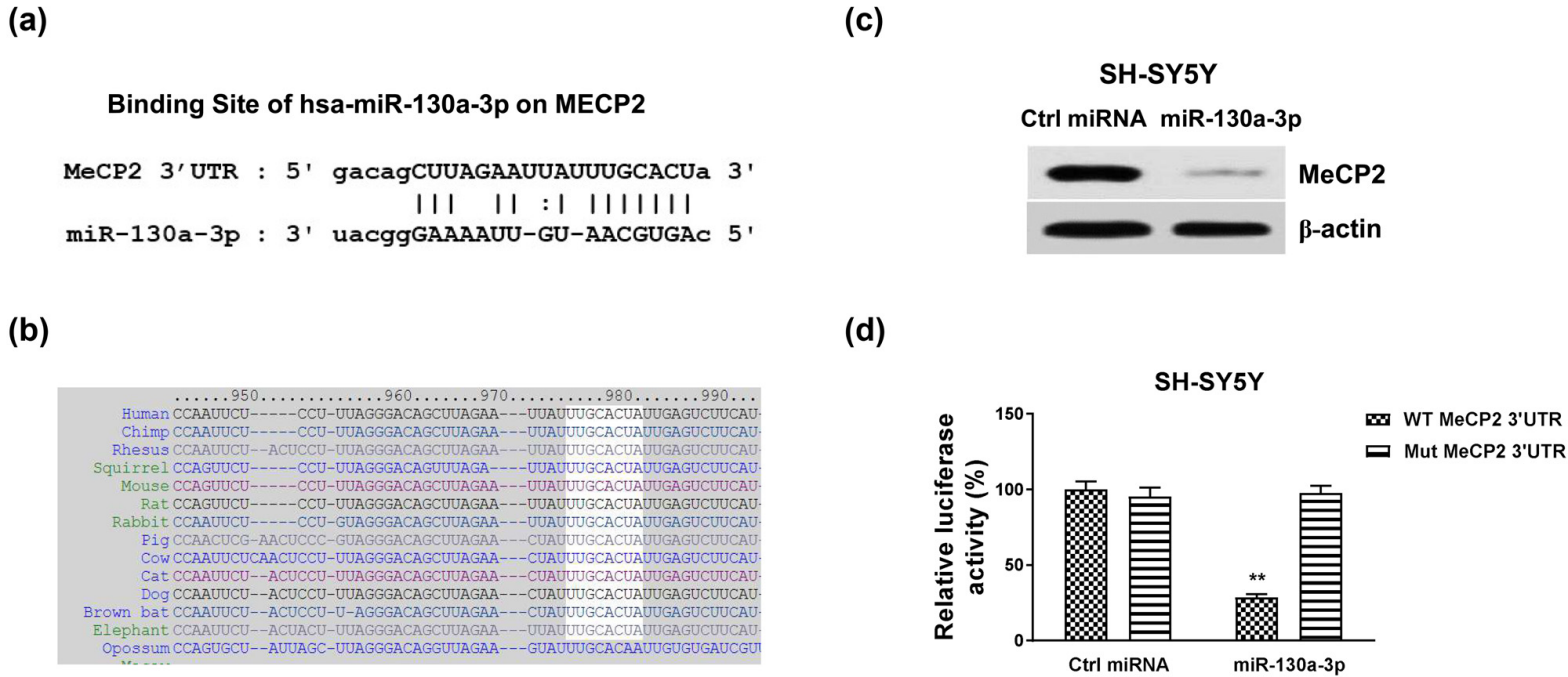
MeCP2 is directly targeted by miR-130a-3p. (a) Bioinformatics analysis of potential binding of miR-130a-3p on 3′UTR of MeCP2. (b) The conserved binding sites of miR-130a-3p on 3′UTR of MeCP2 in multiple species. (c) SH-SY5Y cells were transfected with control or miR-130a-3p for 48 h; the protein expression of MeCP2 was detected by western blot. (d) Dual-luciferase reporter assay was performed in SH-SY5Y cells with co-transfection of WT-MeCP2 or Mut-MeCP2 with control miRNA, or miR-130a-3p. **p < 0.01.

Since miRNAs function via targeting mRNA 3′UTR to downregulate target mRNA expressions [20], we performed miRNA–mRNA interaction analysis from the non-coding RNA database, starBase 2.0. Interestingly, the 3′UTR of MeCP2, which is an important molecule in neuron differentiation and neuronal diseases, contains conserved miR-130a-3p binding sites through multiple species (Figure 3a and b). To examine whether miR-130a-3p could suppress protein expression of MeCP2, miR-130a-3p or control miRNA was transfected into SH-SY5Y cells. The results from western blot showed that overexpression of miR-130a-3p effectively blocked MeCP2 protein expression in SH-SY5Y cells (Figure 3d). To validate the direct binding of miR-130a-3p on 3′UTR of MeCP2 mRNA, SH-SY5Y cells were co-transfected with luciferase vector containing WT-MeCP2 3′UTR or the miR-130a-3p binding site mutant MeCP2 3′UTR (Mut-MeCP2) and control miRNA or miR-130a-3p. Luciferase activity of the vector containing WT-MeCP2 was remarkedly blocked by miR-130a-3p (Figure 3d). Expectedly, the luciferase activity of the Mut-MeCP2 vector has no significant change by miR-130a-3p overexpression (Figure 3d). Together, these results demonstrated that miR-130a-3p could directly target 3′UTR of MeCP2.

### 3.4 MeCP2 is induced and promotes the RA-induced neuron differentiation

**Figure 4:**
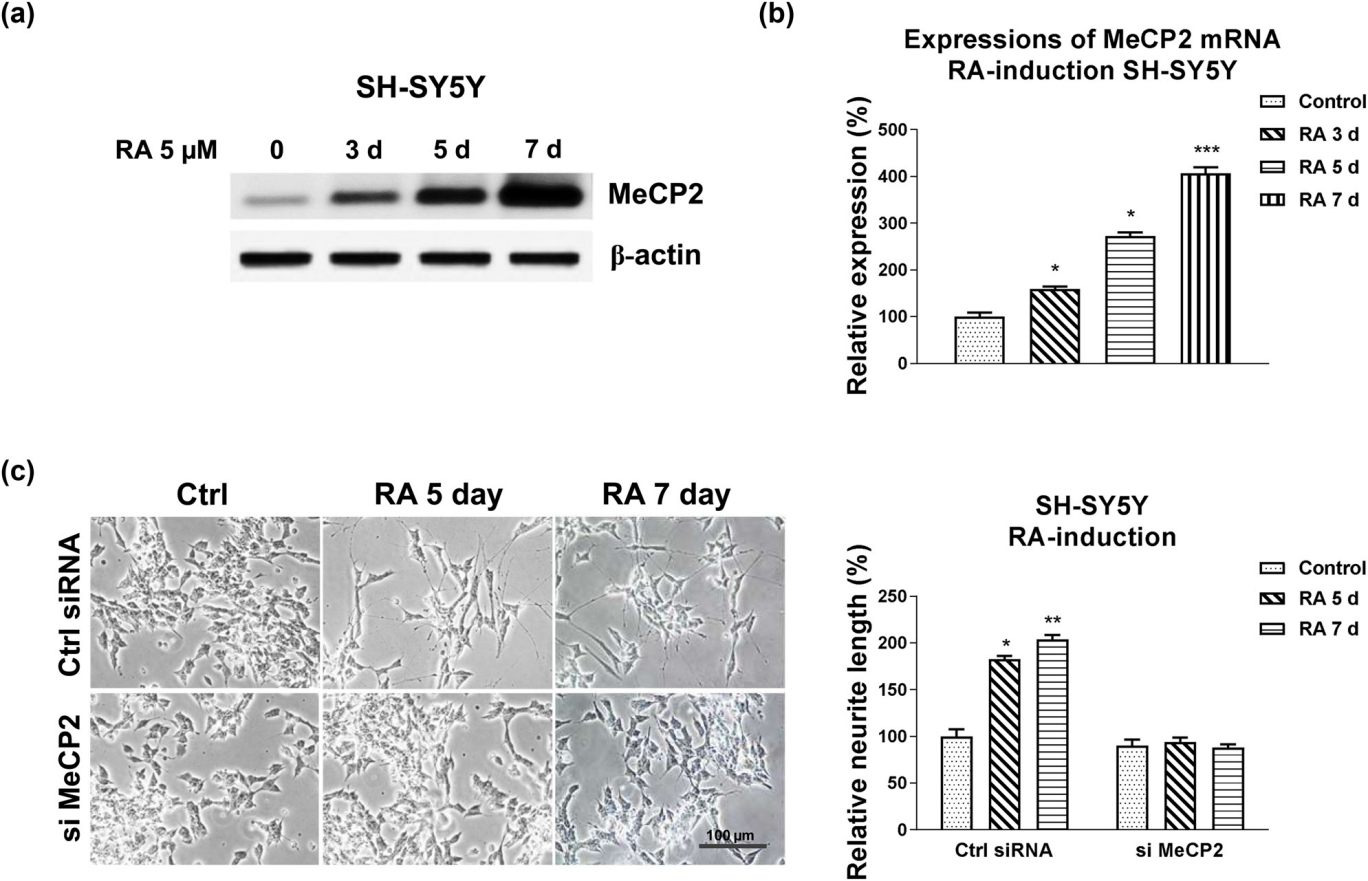
Roles of MeCP2 in the RA-induced neuron differentiation. (a) SH-SY5Y cells were treated with 5 µM RA for 3, 5, and 7 days. Protein and (b) mRNA expressions of MeCP2 were detected. (c) SH-SY5Y cells were transfected with control siRNA or MeCP2 siRNA for 48 h, followed by treatments with 5 µM RA for 5 and 7 days. Cell morphologies were examined under a microscope, and the neurite length was measured. *p < 0.05; **p < 0.01; and ***p < 0.001.

**Figure S4:**
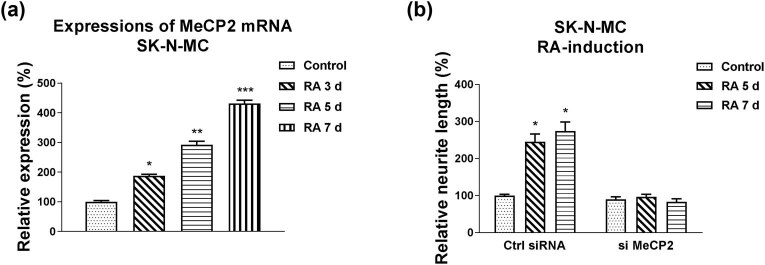
Silencing MeCP2 suppresses RA-induced neuron differentiation. (a) SK-N-MC cells under RA treatments for 3, 5 and 7 days, expressions of MeCP2 were determined by qRT-PCR. (b) The relative neurite length was calculated in SK-N-MC cells without or with MeCP2 silencing under RA treatments for 5 and 7 days. *, *p* < 0.05; **, *p* < 0.01; ***, *p* < 0.001.

We continue to evaluate the roles of MeCP2 in RA-induced neuron differentiation. Under RA treatments, protein and mRNA expressions of MeCP2 were significantly upregulated (Figure 4a and b and Figure S4a). Furthermore, MeCP2 was knocked down by siRNA in SH-SY5Y cells. The results in Figure 4c and Figure S4b illustrate that SH-SY5Y cells with downregulated MeCP2 expression exhibited unchanged neurite extension in response to RA induction compared with control cells, indicating that MeCP2 promotes the process of neuron differentiation.

### 3.5 Restoration of MeCP2 in miR-130a-3p overexpressing SH-SY5Y cells rescues the RA-induced neuron differentiation

**Figure 5:**
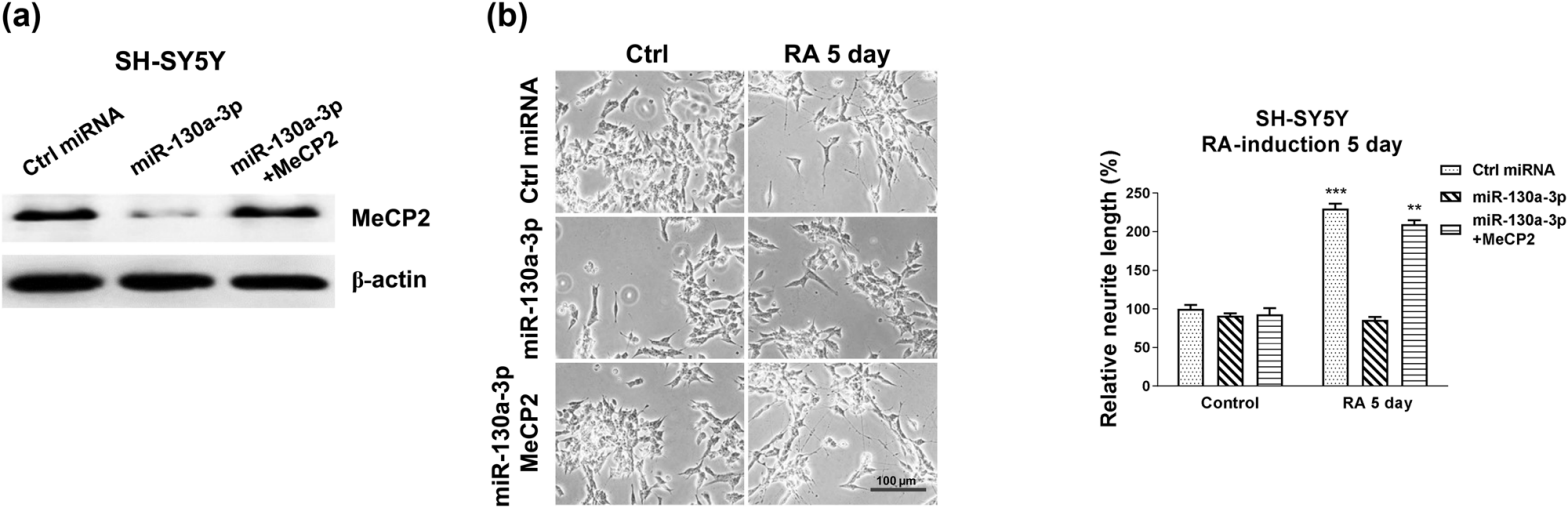
Restoration of MeCP2 rescues the miR-130a-3p suppressed neuron differentiation. (a) SH-SY5Y cells were transfected with control, miR-130a-3p alone, or with MeCP2 overexpression plasmid for 48 h; protein expression of MeCP2 was determined by western blot. (b) The above transfected cells were treated with 5 µM RA for 5 and 7 days. Cell morphologies were examined under a microscope, and the neurite length was measured. *p < 0.05 and **p < 0.01.

Rescue experiments were performed to validate whether miR-130a-3p blocked the RA-induced neuron differentiation through targeting MeCP2. SH-SY5Y cells were transfected with control miRNAs, miR-130a-3p alone or with MeCP2 overexpression plasmid. Co-transfection of miR-130a-3p with MeCP2 successfully recovered MeCP2 protein levels compared with miR-130a-3p overexpression cells (Figure 5a). Moreover, the expected results demonstrated that SH-SY5Y cells with the restoration of MeCP2 rescued the RA-induced neuron differentiation phenotype (Figure 5b). These rescue experiments further confirmed that miR-130a-3p directly targets MeCP2 to inhibit neuron differentiation.

### 3.6 HAGLR promoted neuron differentiation by targeting the miR-130a-3p-MeCP2 axis

**Figure 6:**
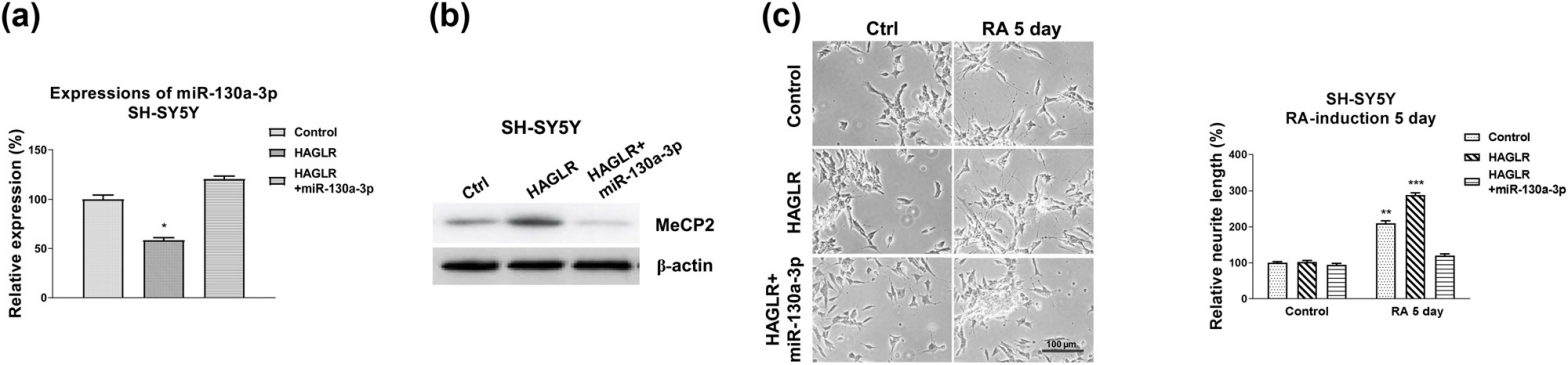
The HAGLR-miR-130a-3p-MeCP2 axis in neuron differentiation. (a) SH-SY5Y cells were transfected with control, HAGLR alone, or with miR-130a-3p for 48 h; expression of miR-130a-3p was detected by qRT-PCR and (b) protein expression of MeCP2 was determined by western blot. (c) The above transfected cells were treated with 5 µM RA for 5 and 7 days. Cell morphologies were examined under a microscope, and the neurite length was measured. *p < 0.05 and **p < 0.01.

Finally, we assessed whether neuron differentiation was regulated by the HAGLR-miR-130a-3p-MeCP2 axis. SH-SY5Y cells were transfected with control, HAGLR overexpression plasmid alone, or with miR-130a-3p. qRT-PCR and western blot results demonstrated that co-transfection of HAGLR with miR-130a-3p successfully restored miR-130a-3p and MeCP2 protein levels compared with HAGLR overexpressing cells (Figure 6a and b). As expected, SH-SY5Y cells with co-transfection of HAGLR and miR-130a-3p re-suppressed the RA-induced neuron differentiation phenotype compared with that from HAGLR overexpressing cells (Figure 6c). In summary, these mechanism rescue experiments consolidated a HAGLR-miR-130a-3p-MeCP2 axis that contributes to the processes of neuron differentiation.

## 4 Discussion

PD is one of the most prevalent neurodegenerative diseases worldwide [1,2]. Currently, the molecular mechanisms underlying PD and the effective disease-modifying approaches are not fully understood. The human neuroblastoma SH-SY5Y cell line is widely studied for investigating the molecular and cellular mechanisms underlying PD pathogenesis and progression since it synthesizes both DA and NA [4]. Moreover, although the SH-SY5Y cell line displays a number of cancerous characteristics, most genes regulations and signal pathways in PD pathogenesis are intact [4]. It is difficult to obtain and maintain human dopaminergic neurons as primary cells, which are mainly affected in PD [4]. Thus, the neuroblastoma SH-SY5Y cell line is a widely used *in vitro* model for PD research. Currently, the biological roles of lncRNA HAGLR in neuronal diseases remain unclear. In this study, we first reported the non-coding RNA-regulated neuron differentiation using SH-SY5Y cells as an *in vitro* model. Under RA induction, we detected that HAGLR was significantly upregulated in SH-SY5Y cells from both microArray analysis and qPCR results. In addition, silencing HAGLR effectively blocked the RA-induced SH-SY5Y differentiation based on the observations that the neurite extension of SH-SY5Y cells with lower HAGLR expression showed little response to RA treatments, suggesting an essential role of HAGLR in SH-SY5Y cell differentiation.

Accumulating studies demonstrated that miRNAs play regulatory roles in neuron development and neuronal diseases [20]. Moreover, the lncRNA–miRNA interaction has been shown to control vital functions during the molecular and cellular processes of human malignancies, including neuronal diseases [8,9]. A recent study reported that miR-130a-3p regulates VEGFR-2 expression in sensory and motor neurons during development [22]. Moreover, the miR-130a-3p/DAPK1 axis was known to regulate the pathophysiology of neonatal hypoxic-ischemia encephalopathy [23], suggesting miR-130-3p to be a potential therapeutic target for the hypoxic ischemia encephalopathy treatment. However, the miR-130a-3p-mediated neuron differentiation has not been investigated. Here, we show that miR-130a-3p was remarkedly downregulated in SH-SY5Y cells under RA induction. Overexpression of miR-130a-3p inhibited the morphological changes of SH-SY5Y cells under RA treatments. Bioinformatics analysis revealed that HAGLR contains miR-130a-3p binding sites. The predicted ceRNA network was further validated by the RNA pull-down assay and luciferase assay.

MeCP2 is the most abundant methyl-DNA binding domain family member in the adult brain and is tightly correlated with diverse neuronal processes during neurodevelopment [15]. It was known that mutations in the MeCP2 gene cause Rett syndrome (RTT), a neurologic condition affecting primarily young girls [16]. Interestingly, girls with RTT exhibiting motor deficits showed similar phenotypes to those in PD [24], suggesting MeCP2 is involved in the defects of the nigrostriatal pathway. A recent study used the 6-hydroxydopamine-induced human neuroblastoma cell (SH-SY5Y cell) injury as a cell model of PD [25]. They described that overexpression of MeCP2 was able to ameliorate the 6-hydroxydopamine-induced apoptosis of SH-SY5Y cells, suggesting that MeCP2 is a potential therapeutic target for the treatment of PD [25]. Currently, the precise roles and molecular mechanisms of MeCP2 in PD have not been elucidated. Here, we described that the expressions of MeCP2 were significantly upregulated in SH-SY5Y cells by RA induction. One advantage of this study is that we illustrated the direct binding of miR-130a-3p on 3′UTR of MeCP2 in SH-SY5Y cells. Furthermore, the rescue experiments validated that the miR-130a-3p-inhibited SH-SY5Y cell differentiation was by direct targeting of MeCP2. Thus, it was possible to target the above signaling pathways in neuronal disease to improve the clinical therapeutic outcomes. These strategies still have limitations owing to the fact that the neuroblastoma cell line is not purely neuron cells since it is oncogenically transformed with catecholaminergic, resulting in different physiological characteristics compared to those from the normal DAergic neuronal features. In addition, the above *in vitro* signaling pathway needs to be verified in animal models.

In summary, this study reports a non-coding RNA-based molecular mechanism for the PD using SH-SY5Y cells as an *in vitro* model. HAGLR positively regulates SH-SY5Y cell differentiation, which is induced by RA via targeting the miR-130a-3p-MeCP2 axis, contributing to an extensive understanding of the pathogenesis and progression of PD.
